# Fractal Analysis of Intramuscular Adipose Tissue on CT Serves as a Novel Imaging Biomarker for Metabolic Syndrome

**DOI:** 10.7150/ijms.126142

**Published:** 2026-03-25

**Authors:** Bowen Hou, Zheng Ran, Jinhan Qiao, Yitong Li, Zhongyichen Huang, Xiaolong Luo, Xiaoming Li

**Affiliations:** 1 Department of Radiology, Tongji Hospital, Tongji Medical College, Huazhong, University of Science and Technology, Wuhan, Hubei Province, China.; 2 Department of MR Imaging, The First Affiliated Hospital of Zhengzhou University, Zhengzhou, China.

**Keywords:** Fractal analysis, Body compositions, Metabolic syndrome, Computed tomography

## Abstract

**Objectives:**

Metabolic Syndrome (MetS) is a cluster of metabolic risk factors, which elevate the risk of cardiovascular diseases and mortality. Body composition, especially the muscle and adipose tissue, plays a critical role in MetS development. The objectives were to explore fractal analysis to quantify the spatial distribution pattern of body composition from computed tomography (CT) and combine with clinical data to develop and validate a diagnostic model for MetS.

**Methods:**

In this two-center retrospective study, participants were classified into MetS and control groups based on International Diabetes Federation criteria. Clinical and CT images of lower extremities were analyzed. A 3D box-counting fractal analysis on CT images was applied to assess the microstructural complexity of body compositions. Feature selection involved univariate analysis, variance inflation factor assessment and multivariate logistic regression. Model performance was evaluated using receiver operating characteristic, decision curve analysis, and calibration curves.

**Results:**

The cohort included 184 subjects from center 1 (split 7:3 into training/internal test sets) and 74 from center 2 for external validation. The final combined model identified five independent predictors: triglyceride (odds ratio (OR) = 2.136), history of diabetes (OR = 7.774), fractal dimension of intramuscular adipose tissue (IMAT) (OR = 3.100), and IMAT multifractal range (OR = 3.613), IMAT/muscle (OR = 1.927). Model combined clinical and radiological features demonstrated robust discrimination, with area under the curves of 0.932 (training), 0.861(test) and 0.855 (validation).

**Conclusion:**

Fractal properties of IMAT derived from CT scans are potent, non-invasive biomarkers for MetS. A diagnostic model integrating radiological features with clinical factors provides excellent, externally validated performance for MetS identification.

## Introduction

Metabolic syndrome (MetS) consists of a cluster of metabolic dysregulations (central obesity, hypertension and dyslipidemia) for cardiovascular disease and mortality, which is a major challenge to clinical practice and public health 1, 2. With its prevalence rising globally, MetS imposes a substantial public medical burden, underscoring the need to elucidate its underlying mechanisms and achieve the early identification and accurate diagnosis.

Body composition plays a critical role in the development of MetS, which is frequently accompanied by an altered balance between skeletal muscle and adipose tissue 3. This imbalance extends not only simple quantitative changes but encompasses a pathological disruption in the endocrine functions of these two metabolic tissues 4, 5. Skeletal muscle, accounting for up to 40% of adult body weight, is a key regulator of systemic energy homeostasis 6, 7. Subcutaneous adipose tissue (SAT) serves as the body's primary site for lipid storage, yet it is also a metabolically active organ with important endocrine properties. However, impairment in either its expandability or its endocrine function can precipitate metabolic dysregulation. Ectopic fat depots exert depot-specific metabolic effects 8. Intermuscular adipose tissue (IMAT) is closely associated with metabolic dysfunction and exhibits unique metabolic properties that differentiate it from other fat depots 9, 10. The muscle and adipose tissue of the lower extremities constitute a major metabolic compartment and serve as a representative indicator of whole-body metabolism 11. Previous studies have largely focused on the quantity of body composition 12, 13, but these conventional metrics fail to capture the spatial heterogeneity of fat infiltration or muscle distribution.

Fractal analysis is a well-established method based on scale invariance and has been increasingly applied to medical imaging for quantifying structural complexity and spatial distribution patterns 14, 15. By characterizing the geometric intricacy of different organs in three dimensions, fractal parameters can effectively reflect histological heterogeneity and spatial organization 16, 17. Fractal-derived indices: fractal dimension (FD) quantifies the overall complexity and space-filling capacity of tissues 18; lacunarity characterizes the heterogeneity and texture of its spatial pattern 19. The efficacy of these indices in revealing subtle pathological variations is well demonstrated in analyses of trabeculated myocardium 20 and acute-stroke perfusion defects 21. By providing insights into tissue microarchitecture under pathological conditions, fractal analysis represents a powerful tool for quantitatively assessing the complex spatial organization of body compositions, with particular relevance to the distribution of muscle and adipose tissues.

In this study, the objectives were to explore fractal analysis to quantify the spatial distribution pattern of body composition and integrate fractal data with the clinical data to develop and validate a diagnostic model for MetS.

## Materials and Methods

### Subjects

Clinical data and CT images of subjects were retrieved from the picture archiving and communication system of center 1 (from September 2019 to June 2021) and center 2 (from February 2023 to June 2024). This multi-center study was conducted with the approval of institutional review board and the requirement for written informed consent was waived due to its retrospective design.

The inclusion criteria for the MetS group were as follows: Age ≥ 18 years, diagnosis of MetS based on International Diabetes Federation criteria^22^, ^23^: Ethnicity-specific waist circumference or a body mass index (BMI) exceeding 30 kg/m², combined with at least two additional criteria, including fasting plasma glucose ≥ 5.6 mmol/L or previously diagnosed type 2 diabetes mellitus, hypertension (systolic/diastolic blood pressure ≥ 130/80 mmHg) or ongoing antihypertensive treatment, plasma triglycerides ≥ 1.7 mmol/L or therapy for hypertriglyceridemia, and reduced high-density lipoprotein cholesterol (HDL-C) levels (< 1.03 mmol/L for men and < 1.29 mmol/L for women) or specific treatment for low HDL-C. Availability of lower extremities CT images with good image quality, complete laboratory examinations and medical history recording. Subjects were excluded if they had a history of malignant tumor, systemic infection, severe trauma or surgery of the lower extremity, inadequate image quality or incomplete medical records. Following the same criteria, control subjects in good health were identified and selected from the same databases.

**Figure 1:**
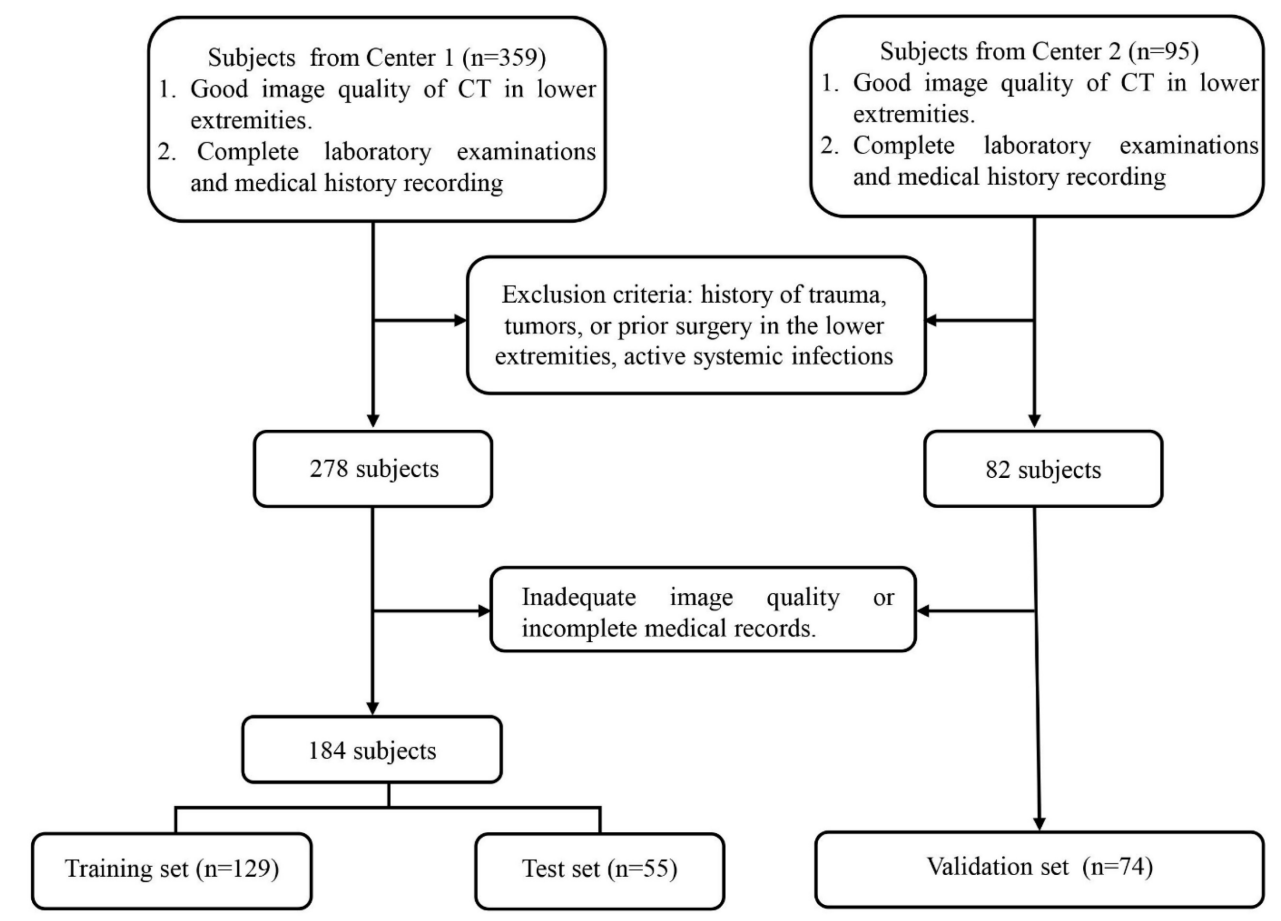
The flowchart of subject recruitment.

Subjects from center 1 were randomly split into training (70 %) and internal test (30 %) sets; all subjects from center 2 served as the external validation set. The participant recruitment flowchart is presented in Figure 1.

### Image acquisition and preprocessing

CT images were acquired primarily using Toshiba and GE scanners, with a tube voltage of 120 kVp and tube current ranging from 120 to 300 mA. The scan range covered the lower extremities, defined from the inferior margin of tibia to the lesser trochanter of the femur.

Following acquisition, all images were anonymized and constructed at the uniform thickness of 1mm with standard kernel. To minimize inter-scanner variability and reduce noise, Gaussian filtering was applied using Python 3.13. Additionally, Hounsfield unit (HU) normalization was performed using skeletal muscle as internal reference to ensure quantitative consistency across different scanners.

### Volumetric body compositions analysis

**Figure 2:**
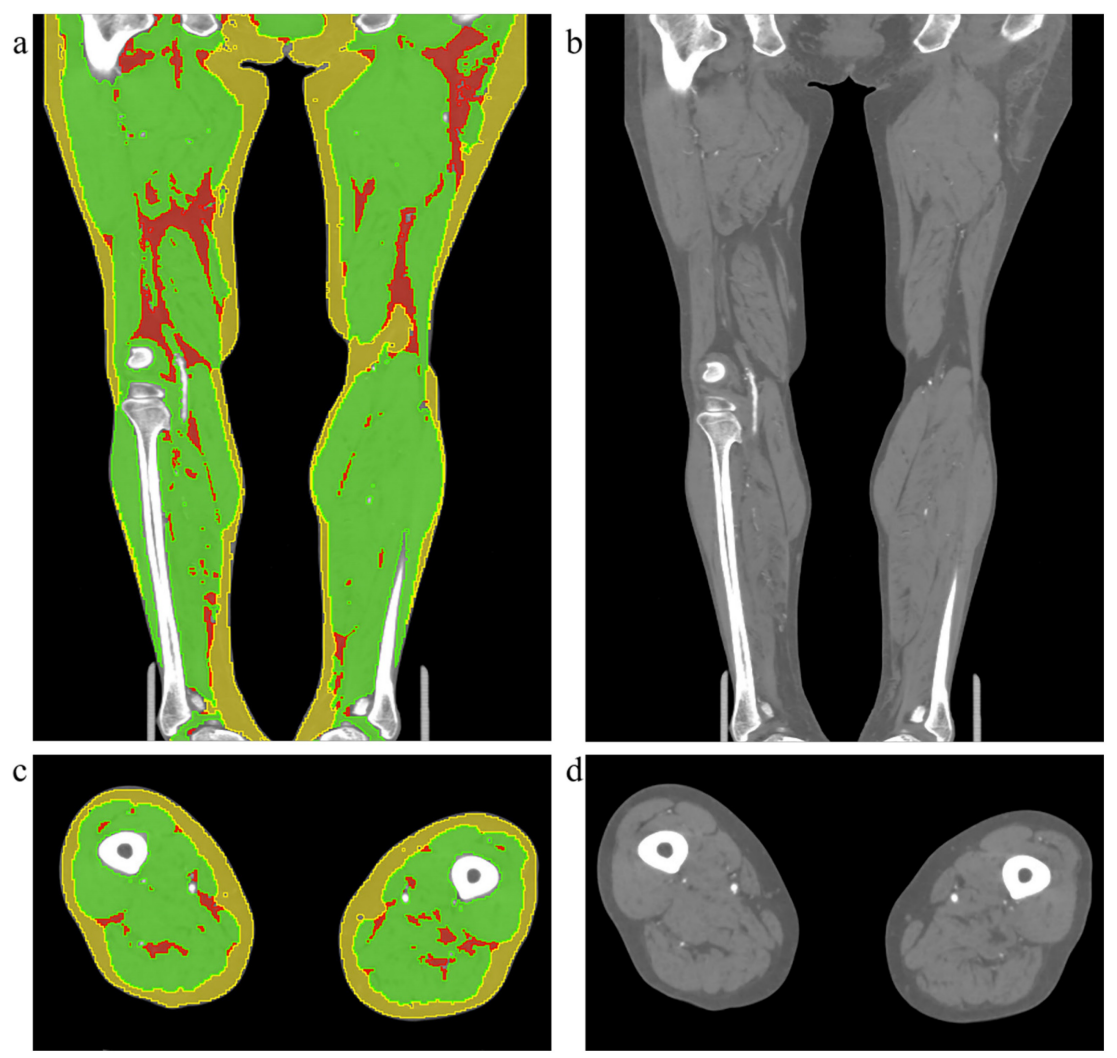
Illustration of the original CT images and body compositions segmentation. The left column (a, c) shows examples of body composition segmentation in the coronal and axial planes, respectively. The right column (b, d) displays the corresponding original CT images. The yellow region represents subcutaneous adipose tissue, the green region indicates muscle, and the red region indicates intermuscular adipose tissue.

Body composition segmentation of lower extremities was performed in 3D-Slicer 5.9.0 by a single radiologist using a semi-automated workflow. Regions of interest corresponding to SAT, skeletal muscle (SM), and IMAT were delineated based on anatomical boundaries and validated HU thresholds. The segmentation was independently performed by two radiologists, and inter-observer consistency was subsequently evaluated. To assess intra-observer reliability, one of the radiologists repeated the same segmentation procedure after an interval of one month. The illustration of body composition is displayed in Figure 2. Volumes of each tissue were calculated from the segmented masks. To reduce the variations in body size, all volumetric measurements were normalized by the cube of height (m^3^), yielding the following standardized indices: Skeletal Muscle Index (SMI), Intramuscular Adipose Tissue Index (IMATI), Subcutaneous Adipose Tissue Index (SATI) and the ratios between tissues were also calculated : IMAT-to-Muscle Ratio (IMR = IMATI / SMI), Muscle-to-SAT Ratio (MSR = SMI / SATI), and IMAT-to-SAT Ratio (ISR = IMATI / SATI).

### Fractal analysis

3D Box-counting fractal analysis was used to quantify microstructural alterations in the spatial distribution of body composition. Based on the previously generated tissue masks (SAT, SM and IMAT), key fractal parameters of FD, lacunarity (Λ) and multifractal range were computed with the dedicated Python package (fractalysis). The core computations of fractal indices were as follows:

#### 1. Fractal dimension

log *N*(ε) = FD · log(1/ε) + *c,*

ε = box side length (pixels), *N*(ε) = number of boxes containing ≥ 1 foreground pixel, and *c* = intercept.

#### 2. Lacunarity (Λ)

Λ(ε) = σ²[*M*(ε)] / (μ[*M*(ε)])²

Μ(*M*(ε)) = mean pixel mass inside occupied boxes and σ²[*M*(ε)] = corresponding variance, averaged over all scales.

#### 3. Multifractal range

ΔFD = |slope_lower - slope_upper|

slope_lower and slope_upper are the slopes of the log-log regression over the first and second halves of box sizes, respectively.

The fractal indices were computed separately for the SM, SAT, and IMAT, with the mean value from both legs used for the final analysis.

### Statistical analyses

Demographic, clinical, volumetric, and fractal parameters were compared between MetS and control groups. Categorical variables were analyzed using the chi-square test or Fisher's exact test, as appropriate. Normality of continuous variables was assessed using the Shapiro-Wilk test. Group comparisons were performed using the independent Student's t-test for normally distributed data and the Mann-Whitney U test for non-normal data.

Inter- and intra-observer agreement of body compositions segmentation was evaluated using the intraclass correlation coefficient (ICC). The strength of agreement was interpreted based on the following thresholds: ICC values ≤ 0.40 were considered as poor agreement, values between 0.40 and 0.60 indicated moderate agreement, values between 0.60 and 0.80 reflected good agreement, and values greater than 0.80 denoted excellent agreement.

Univariate logistic regression was applied to the training set (data from center 1) to screen for variables potentially associated with MetS. Candidate predictors for multivariable modeling were selected based on a univariate significance level of *p* < 0.1. To ensure model stability and mitigate the influence of multicollinearity, variance inflation factors (VIF) and Spearman correlation analysis were calculated for all candidate variables. Any variable exhibiting a VIF greater than 10 and r greater than 0.8 (indicating severe multicollinearity) was sequentially removed prior to constructing the final model. The retained variables were then entered into a multivariable logistic regression framework to identify independent predictors of MetS.

The diagnostic performance of the final logistic regression model was evaluated using several metrics: discriminative ability was quantified by the area under the receiver operating characteristic (ROC) curve, clinical utility was assessed via decision curve analysis (DCA), and the agreement between predicted probabilities and observed outcomes was examined using calibration curves. All statistical analyses were conducted using SPSS (SPSS Inc., Armonk, NY, USA), Python 3.13, and R software (version 4.5.1).

## Results

### Patient Characteristics and Radiological analysis

A total of 258 subjects were included in the final analysis. The cohort was derived from two centers: 184 subjects (93 with MetS and 91 controls) from center 1 were randomly divided into training (n = 129) and internal test (n = 55) sets at a 7:3 ratio; 74 subjects (35 with MetS and 39 controls) from center 2 constituted the external validation set. Demographic and clinical characteristics stratified by sex and MetS status are presented in Table 1.

As expected, compared to the control group, subjects in the MetS group showed a significantly higher prevalence of cardiometabolic risk factors, including diabetes and hypertension. The results of the volumetric body composition analysis and fractal analysis of tissue distribution are also detailed in Table 1.

### Intra- and inter-observer agreement of body composition segmentation

The intra- and inter-observer ICC analyses for body composition segmentation are shown in Table 2. All inter- and intra-observer ICC values ranged 0.900-1.000, indicating excellent agreement. Among all the body compositions, muscle showed higher intra-observer and inter-observer ICC values, with the highest ICC value of 0.992 (95% confidence interval (CI): 0.988-0.995), and 0.983 (95% CI: 0.975-0.989). IMAT displayed lower intra-observer and inter-observer ICC values with 0.950 (95% CI: 0.926-0.966) and 0.946 (95% CI: 0.918-0.965).

### Variables Selection and Model development

Twenty-eight demographic, laboratory and radiological variables were evaluated. Univariate logistic regression of training set identified eight clinical predictors associated with MetS: history of diabetes (Odds ratio (OR) = 5.587, *p* < 0.001), alcohol consumption (OR = 3.744, *p* = 0.004), waist circumference (OR = 1.767, *p* = 0.004), smoking (OR = 2.891, *p* = 0.008), triglyceride level (OR = 2.174,* p* = 0.001), height (OR = 1.505, *p* = 0.027), and weight (OR = 1.610, *p* = 0.012). Three radiological features of IMAT the was associated with MetS: IMR (OR = 2.644, *p* < 0.001), IMAT FD (OR = 2.274, *p* < 0.001) and IMAT multifractal range (OR = 2.739, *p* < 0.001). These results are summarized in Table 3.

After assessing multicollinearity among the candidate variables, three models were built with the same training data: Clinical model (with clinical factors), Radiological model (with IMR + IMAT FD + IMAT multifractal range); Combined model (with clinical and IMAT features). The final combined model retained five independent contributors: triglyceride level (OR = 2.136, *p* = 0.019), diabetes history (OR = 7.774, *p* = 0.002), IMR (OR = 1.927, *p* = 0.043), IMAT FD (OR = 3.100, *p* = 0.001), and IMAT multifractal range (OR = 3.613, *p* < 0.001). The corresponding results are displayed in Table 4.

### Feature Importance and Model Performance

**Figure 3:**
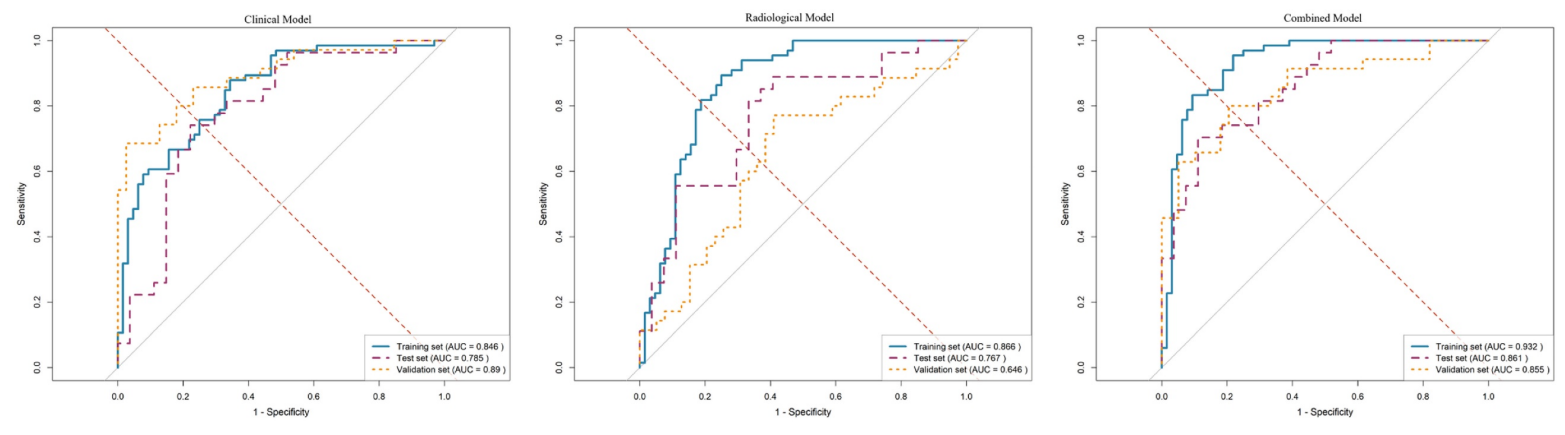
ROC curves of different models in training, internal test and validation sets. AUC: Area under the curve.

The performance metrics, including the area under the curve (AUC), sensitivity, specificity, and accuracy of all models, are detailed in Table 5. As illustrated by the ROC curves in Figure 3, the combined model demonstrated superior discriminative ability compared to both the clinical model (AUC: 0.846 in training set, 0.785 in test set, 0.890 in validation set) and the radiological model (AUC: 0.866 in training set, 0.767 in test set, 0.646 in validation set). Specifically, the combined model achieved AUC values of 0.932 in the training set, 0.861 in the internal test set, and 0.855 in the external validation set. In the validation set, it also yielded a sensitivity of 0.800, a specificity of 0.795, and an overall accuracy of 0.797.

**Figure 4:**
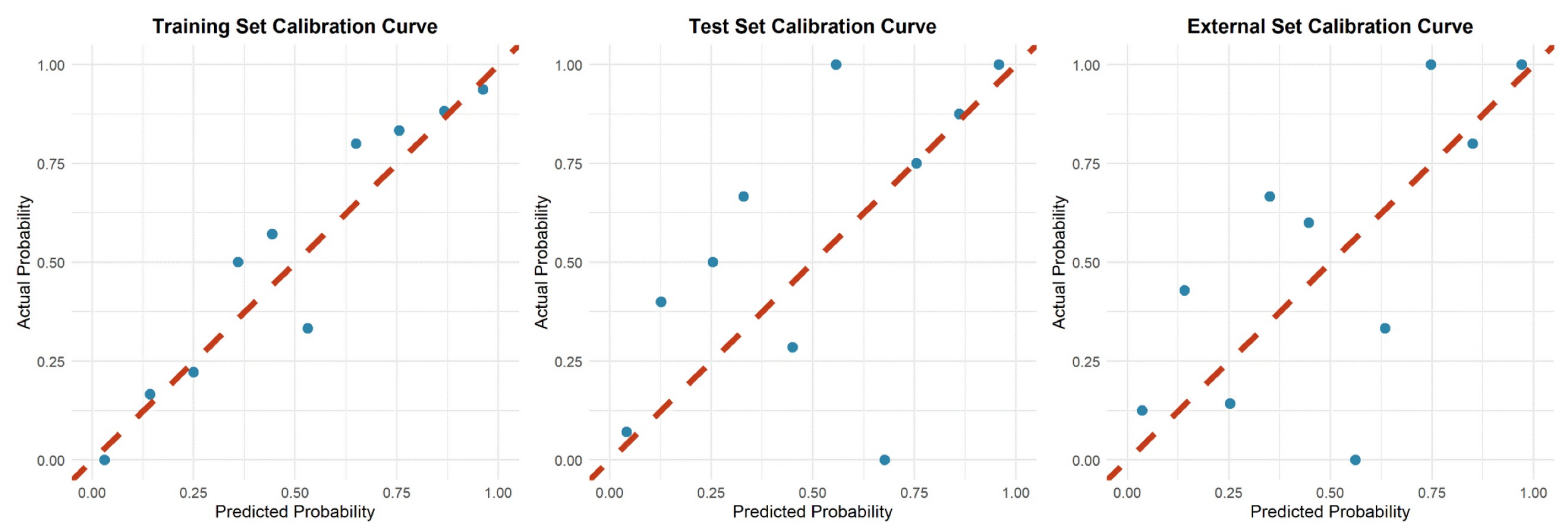
Calibration illustration of combined model in training, internal test and validation sets.

**Figure 5:**
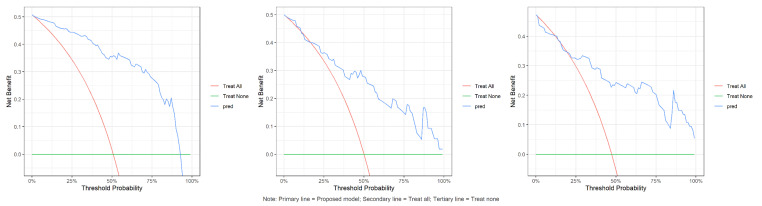
Decision curves of combined model in training, internal test and validation sets. The red curve denotes the "treat all " strategy, the green curve denotes the "treat none" strategy, and the blue curve denotes the net benefit of the combined model in this study. The results demonstrate that the combined model yields a higher net benefit than both extreme strategies across a range of threshold probabilities, indicating its potential clinical utility.

**Figure 6:**
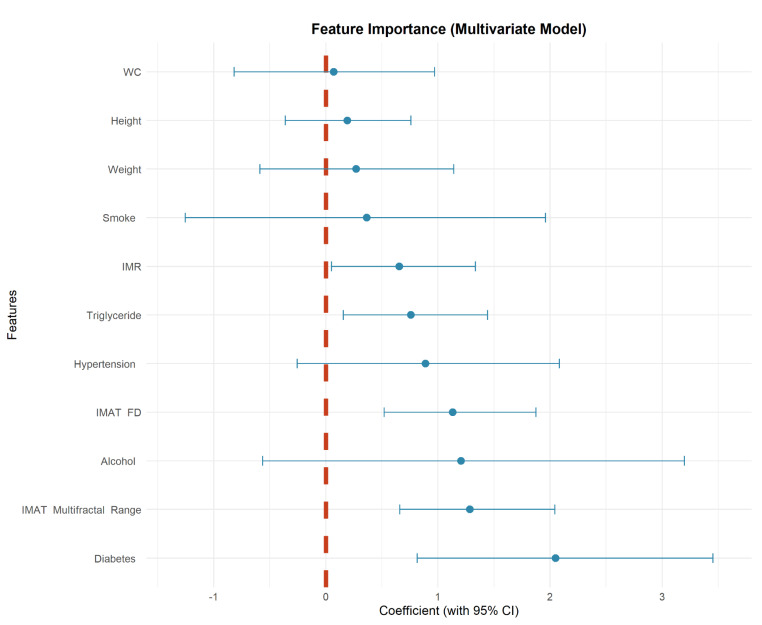
Coefficient forest plot of feature importance in combined model.

Calibration plots indicated good agreement between predictions and observations for the combined model in the test set (Figure 4). As expected, calibration performance slightly decreased in the external validation set due to cohort differences (Figure 4 and Supplementary Materials S1, S2). DCA was performed to evaluate the clinical net benefit of the model. The combined model provided the highest clinical net benefit across a wide range of threshold d probabilities in cohorts (Figure 5 and supplement material S3, 4). Furthermore, a forest plot of the combined model (Figure 6) revealed the five most influential features for MetS diagnosis: history of diabetes, IMAT multifractal range, alcohol consumption, IMAT FD, and hypertension.

## Discussion

In this study, besides conventional volumetric assessment, fractal analysis was employed to evaluate the distribution of body composition in individuals with MetS. Fractal features of IMAT captured the spatial heterogeneity of distribution and were independent predictors of MetS. The final combined model consisted of five variables, including two IMAT fractal features. It achieved an AUC of ≥ 0.85 in both the internal and external validation sets, outperforming the purely clinical and radiological models.

Body composition analysis is increasingly employed in research with multiple modalities available for quantitative evaluation 24, 25. While dual-energy X-ray absorptiometry is widely used for body composition mass evaluation, it is susceptible to variations in hydration status 26. Bioelectrical impedance analysis estimates body composition based on tissue resistance to electrical current; however, its accuracy is easily influenced by an individual's hydration level, food intake, and electrode placement 27. In contrast, CT and magnetic resonance imaging provide cross-sectional visualization of tissues, allowing for precise quantification of specific adipose depots. Although numerous studies have quantified body composition using conventional image-based metrics (e.g., volume or HU values), these approaches typically fail to capture their spatial distribution 28, 29. However, fractal analysis serves as a valuable tool for quantifying structural complexity, irregularity, and spatial patterning 30, 31, offering novel perspectives on quantifying how heterogeneously fat threads through muscle fascia in spatial distribution scale. Fractal parameters quantify the spatial-filling efficiency and geometric intricacy of organs, capturing global patterning properties 32. In comparison, texture features are based on signal processing and statistics, focusing on the statistical relationships among pixels and quantifying local heterogeneity through distributions of gray values 33, 34.

The utility of fractal analysis has been demonstrated across diverse fields, including the assessment of myocardial microvascular ischemia 35, subjective cognitive decline 36, and trabecular bone architecture 37. Its ability to capture microarchitectural changes associated with disease progression underscores its methodological robustness. While fractal parameters offer distinct advantages for quantifying tissue heterogeneity, their application requires specific methodological conditions. These metrics are not specific to any biological process and should be interpreted as general descriptors of microstructural organization rather than direct indicators of specific pathological changes. FD is effective for assessing global complexity but is sensitive to image resolution and segmentation quality 19. Lacunarity captures clustering and spatial distribution, which is valuable for evaluating infiltration patterns, yet it can be influenced by region of interest selection 38. By using high-resolution images and semi-automatic segmentation, we aimed to minimize these technical confounders. The 3D box-counting approach was chosen for its relative simplicity, low dimensionality, and ability to capture overall spatial complexity. Fractal analysis lies in being reproducible quantitative descriptors that may reflect changes in muscle and adipose tissue microstructure changes associated with metabolic dysfunction.

The key finding in this study indicated that the features of IMAT, specifically IMR, FD and multifractal range serve as strong and independent predictors of MetS. This observation aligned with the growing recognition of IMAT as a metabolically active depot that contributes to insulin resistance and systemic inflammation 39, 40. The significant predictive value of both FD and multifractal range indicated that beyond the simple quantitation of tissues, the spatial patterning of IMAT captured unique aspects of metabolic dysregulation. Previous studies on IMAT had predominantly relied on mass or indirect mean muscle attenuation values 41, 42. These conventional metrics, which are grounded in the anatomical definition of IMAT as fat located between muscle compartments and inherently limited in their ability to characterize spatial distribution patterns. Fractal parameters of IMAT (FD and multifractal range) provide a more comprehensive characterization of pathological fat infiltration than volumetric measures alone in this study. The diagnostic model integrating these imaging biomarkers with clinical parameters showed improved discriminatory power and good calibration.

In metabolic diseases, IMAT is not merely a passive fat depot but an active endocrine and inflammatory tissue. Its distribution and microstructural complexity have been linked to insulin resistance, systemic inflammation, and muscle function decline 42. Fractal analysis enables the detection of early, subvisual changes in IMAT morphology on routine CT images, potentially identifying individuals at high risk of metabolic deterioration before overt changes in fat volume or anthropometric measures occur. From a clinical perspective, fractal biomarkers offer a powerful tool for enhancing risk stratification, personalizing prevention, and monitoring therapeutic response. Their integration into practice could improve early diagnosis, prognostic accuracy, and treatment monitoring, thereby advancing toward a more structural and mechanistic understanding of metabolic diseases.

Several limitations must be acknowledged. Although the sample size is sufficient for model development and initial validation, its modest scale and relatively small effective events necessitate future large-scale, multicenter, and multi-ethnic studies to ensure generalizability. Furthermore, the reliance on CT for body composition assessment provides anatomical rather than direct pathological data. Although the fractal alterations are statistically robust, the specific microstructural and biological mechanisms underlying them remain to be fully elucidated in future research.

## Conclusion

In conclusion, fractal properties of IMAT derived CT scans are potent, non-invasive biomarkers for MetS. A diagnostic model integrating radiological features with clinical factors provides excellent, externally validated performance for MetS identification.

## Supplementary Material

Supplementary figures.

## Figures and Tables

**Table 1:** Basic characteristic and radiological features of subjects.

Parameters	Center 1				Center 2			
	MetS (n = 93)		Control (n = 91)		MetS (n = 35)		Control (n = 39)	
Age (y)	Male: 62.19±12.238	Female: 64.71±11.140	Male: 60.32±9.949	Female: 60.87±14.295	Male: 62.462±8.058	Female: 68.143±13.530	Male: 62.235±10.910	Female: 71.417±8.670
Sex (male: female)	58:35		53:38		21:14		28:11	
Weight (kg)	Male: 69.198±10.893	Female: 64.046±10.030	Male: 66.801±9.189	Female: 54.809±9.426	Male: 81.769 ±10.240	Female: 67.536±14.637	Male: 64.221±7.989	Female: 54.708±10.805
Height (m)	Male: 1.665±0.079	Female: 1.651±0.082	Male: 1.647±0.080	Female: 1.606±0.076	Male: 1.738±0.054	Female: 1.630±0.063	Male: 1.700±0.046	Female: 1.640±0.0822
BMI (kg/m2)	Male: 25.042±4.186	Female: 23.636±4.239	Male: 24.755±3.812	Female: 21.291±3.741	Male: 27.096±3.269	Female: 25.249±4.306	Male: 22.209±2.552	Female: 20.233±3.055
Waist circumference (cm)	Male: 86.943±10.469	Female: 88.644±11.032	Male: 85.138±10.210	Female: 78.283±7.819	Male: 95.875 ±5.051	Female: 91.636±7.362	Male: 82.294±7.076	Female: 78.583±8.670
HDL-C (mmol/L)	Male: 1.001±0.312	Female: 1.152±0.262	Male: 0.818±0.200	Female: 0.982±0.327	Male: 0.853±0.228	Female: 0.981±0.279	Male: 1.020±0.256	Female: 1.256 ±0.364
Triglyceride (mmol/L)	Male: 2.181±1.178	Female: 2.132±1.073	Male: 1.258±0.792	Female: 1.399±0.980	Male: 1.831±1.035	Female: 1.471±0.625	Male: 1.234±0.614	Female: 1.391±0.561
Total cholesterol (mmol/L)	Male: 4.404±1.113	Female: 3.969±1.121	Male: 3.836±1.025	Female: 3.928±1.051	Male: 4.176±0.762	Female: 4.113 ±0.835	Male: 3.549±0.840	Female: 3.801 ±1.511
Diabetes	66/93		39/91		35/35		15/39	
Hypertension	55/93		40/91		25/35		14/39	
Smoking	38/93		20/91		16/35		20/39	
Alcohol	26/93		12/91		11/35		7/39	
SMI	2639.360±634.740		2630.854±667.109		3929.871±1419.571		3626.738±891.757	
SATI	2217.515±917.773		2123.907±974.671		3437.541±2001.054		2468.898±1016.150	
IMATI	442.615±174.547		400.794±176.547		784.678±390.374		642.500±291.405	
IMR	0.220±0.072		0.157±0.067		0.207±0.081		0.183±0.081	
ISR	0.221±0.096		0.235±0.195		0.259±0.135		0.295±0.179	
MSR	1.444±0.822		1.583±1.108		1.352±0.678		1.724±0.833	
Muscle FD	1.480±0.045		1.481±0.050		1.426±0.045		1.426±0.046	
Muscle lacunarity	11.834±2.909		11.949±3.179		18.586±4.532		18.992±5.050	
Muscle multifractal range	0.337±0.834		0.328±0.089		0.403±0.079		0.456±0.080	
SAT FD	1.372±0.068		1.377±0.084		1.346±0.064		1.314±0.065	
SAT lacunarity	16.451±4.798		16.491±6.018		22.023±5.575		24.238±6.707	
SAT multifractal range	0.309±0.076		0.304±0.078		0.329±0.067		0.324±0.083	
IMAT FD	1.120±0.037		1.170±0.066		1.166±0.045		1.135±0.068	
IMAT lacunarity	40.549±3.772		38.420±7.105		48.595±8.623		53.263±11.268	
IMAT multifractal range	0.219±0.035		0.186±0.042		0.209±0.062		0.198±0.048	

BMI: Body Mass Index, WC: Waist circumference, HDL-C: High Density Lipoprotein Cholesterol, IMAT: Intramuscular Adipose Tissue, SAT: Subcutaneous Adipose Tissue, SMI: Skeletal Muscle Index, IMATI: Intramuscular Adipose Tissue Index, SATI: Subcutaneous Adipose Tissue Index, IMR=IMAT/SMI, MSR=SMI/SAT, and ISR=IMAT/SAT. FD: fractal dimension.

**Table 2:** Intra- and inter-observer agreement for body composition segmentation.

Parameters	Inter-observer agreement			Intra-observer agreement		
	ICC	95% CI	P	ICC	95% CI	P
SAT	0.948	0.921-0.966	0.000	0.960	0.942-0.973	0.000
IMAT	0.946	0.918-0.965	0.000	0.950	0.926-0.966	0.000
Muscle	0.983	0.975-0.989	0.000	9.992	0.988-0.995	0.000
Total	0.943	0.913-0.962	0.000	0.950	0.926-0.966	0.000

CI: confidence interval, IMAT: Intramuscular Adipose Tissue, SAT: Subcutaneous Adipose Tissue

**Table 3:** Univariate regression analysis of clinical and radiologic features.

Variables	OR	95% CI	P
Age	1.099	0.777-1.563	0.593
Sex	1.057	0.529-2.116	0.875
Weight	1.610	1.121-2.378	0.012^#^
Height	1.505	1.056-2.191	0.027^#^
BMI	1.237	0.874-1.772	0.234
WC	1.767	1.221-2.639	0.004^#^
HDL-C	0.993	0.698-1.411	0.967
Triglyceride	2.174	1.411-3.639	0.001^#^
Total cholesterol	0.783	0.545-1.111	0.176
Diabetes	5.587	2.607-12.589	0.000^#^
Hypertension	1.979	0.990-4.012	0.055^#^
Smoking	2.891	1.346-6.466	0.008^#^
Alcohol	3.744	1.579-9.686	0.004^#^
SMI	0.999	0.705-1.416	0.997
SATI	1.113	0.788-1.587	0.544
IMATI	1.337	0.938-1.969	0.121
IMR	2.644	1.731-4.258	0.000^#^
ISR	0.887	0.584-1.264	0.518
MSR	0.848	0.576-1.203	0.367
Muscle FD	1.015	0.717-1.437	0.935
Muscle lacunarity	1.001	0.707-1.419	0.994
Muscle multifractal range	1.026	0.725-1.455	0.886
SAT FD	1.037	0.732-1.472	0.837
SAT lacunarity	0.941	0.662-1.332	0.730
SAT multifractal range	1.071	0.758-1.519	0.698
IMAT FD	2.274	1.506-3.613	0.000^#^
IMAT lacunarity	1.316	0.927-1.906	0.133
IMAT multifractal range	2.739	1.801-4.380	0.000^#^

# represents p < 0.1. BMI: Body Mass Index, WC: Waist circumference, HDL-C: High Density Lipoprotein Cholesterol, IMAT: Intramuscular Adipose Tissue, SAT: Subcutaneous Adipose Tissue, SMI: Skeletal Muscle Index, IMATI: Intramuscular Adipose Tissue Index, SATI: Subcutaneous Adipose Tissue Index, IMR=IMAT/SMI, MSR=SMI/SAT, and ISR=IMAT/SAT. FD: Fractal Dimension

**Table 4:** Multivariate regression analysis of clinical and radiologic features.

Variables	OR	95% CI	P
WC	1.073	0.442-2.640	0.876
Weight	1.312	0.555-3.131	0.534
Height	1.211	0.697-2.136	0.497
Triglyceride	2.136	1.169-4.235	0.019^*^
Diabetes	7.774	2.258-31.606	0.002^*^
Hypertension	2.431	0.775-8.026	0.132
Smoking	1.440	0.285-7.092	0.652
Alcohol	3.345	0.569-24.524	0.203
IMR	1.927	1.052-3.798	0.043^*^
IMAT FD	3.100	1.685-6.510	0.001^*^
IMAT Multifractal Range	3.613	1.934-7.716	0.000^*^

* represents p < 0.05. WC: Waist circumference, IMAT: Intramuscular Adipose Tissue, IMR=IMAT/SMI, FD: Fractal Dimension

**Table 5:** Performance metrics of the clinical model, radiological model, and combined model in different cohorts.

Model	AUC	Sensitivity	Specificity	Accuracy	PPV	NPV
**Clinical model**						
Training set	0.846	0.879	0.656	0.769	0.725	0.840
Test set	0.785	0.741	0.778	0.759	0.769	0.750
Validation set	0.890	0.686	0.974	0.838	0.96	0.776
**Radiologic model**						
Training set	0.866	0.894	0.750	0.823	0.787	0.873
Test set	0.767	0.889	0.593	0.741	0.686	0.842
Validation set	0.646	0.771	0.590	0.676	0.628	0.742
**Combined Model**						
Training set	0.932	0.833	0.906	0.869	0.902	0.841
Test set	0.861	0.704	0.889	0.796	0.864	0.750
Validation set	0.855	0.800	0.795	0.797	0.778	0.816

AUC: area under the curve, PPV: positive predictive value, NPV: negative predictive value

## Abbreviations

AUC: Area under the curve
BMI: Body mass index
CI: Confidence interval
CT: Computed tomography
DCA: Decision curve analysis
FD: Fractal dimension
HDL-C: High density lipoprotein cholesterol
HU: Hounsfiled unit
ICC: Intraclass Correlation Coefficient
IMAT: Intramuscular adipose tissue
IMATI: Extracellular lipid index
IMR: Ratio of intramuscular adipose tissue and muscle
ISR: Ratio of intramuscular adipose tissue and subcutaneous adipose tissue
MetS: Metabolic syndrome
MSR: Ratio of muscle and subcutaneous adipose tissue
OR: Odds ratio
PACS: Picture archiving and communication system
ROC: Receiver operating characteristic
SAT: Subcutaneous adipose tissue
SATI: Subcutaneous Adipose Tissue Index
SM: Skeletal muscle
SMI: Skeletal Muscle Index
WC: Waist circumference
