# Supplementary material for: Fractal Analysis of Intramuscular Adipose Tissue on CT Serves as a Novel Imaging Biomarker for Metabolic Syndrome
Source: Int J Med Sci. 2026 Mar 25;23(5):1645–55. doi: 10.7150/ijms.126142 (PMC13133887; doi:10.7150/ijms.126142)

S1 Calibration performance of clinical model in training, test and validation sets.

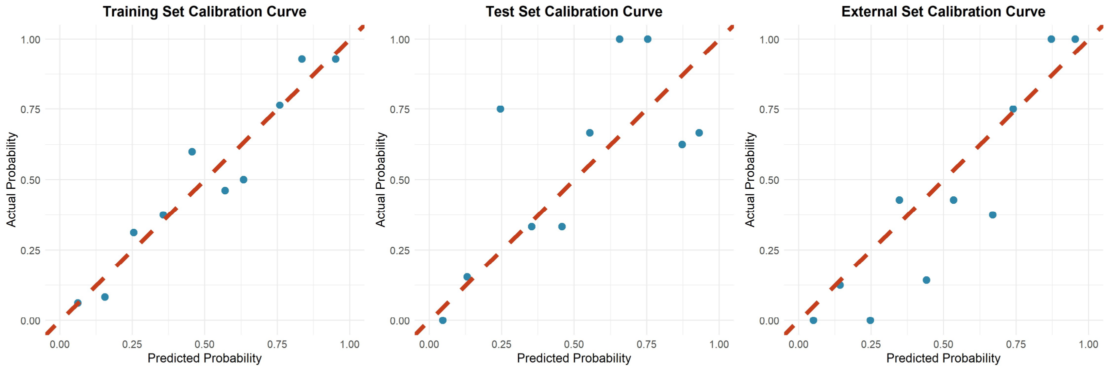

S2 Calibration performance of radiological model in training, test and validation sets.

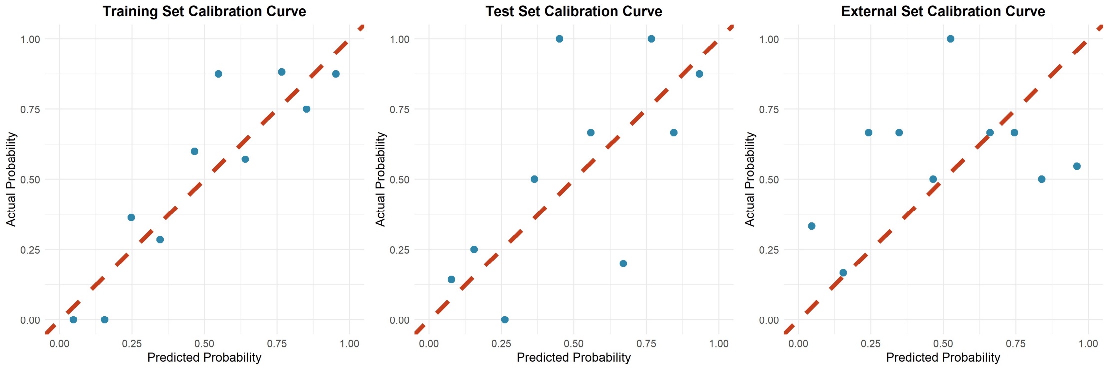

S3 Decision curve analysis of clinical model in train, set and validation sets.

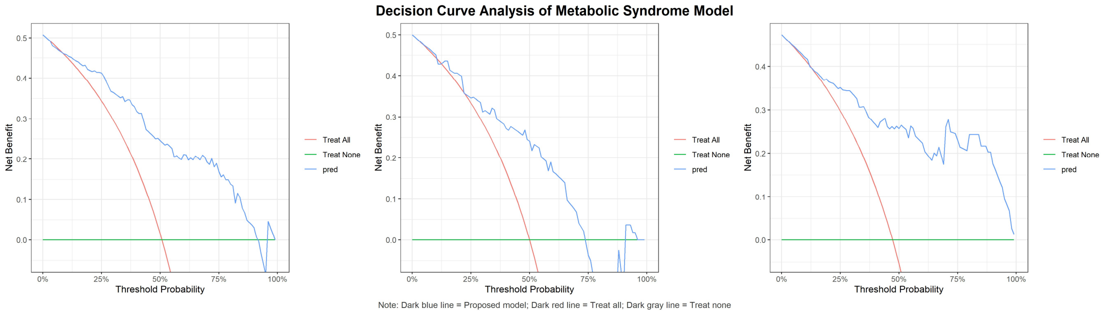

S4 Decision curve analysis of radiological model in train, set and validation sets.

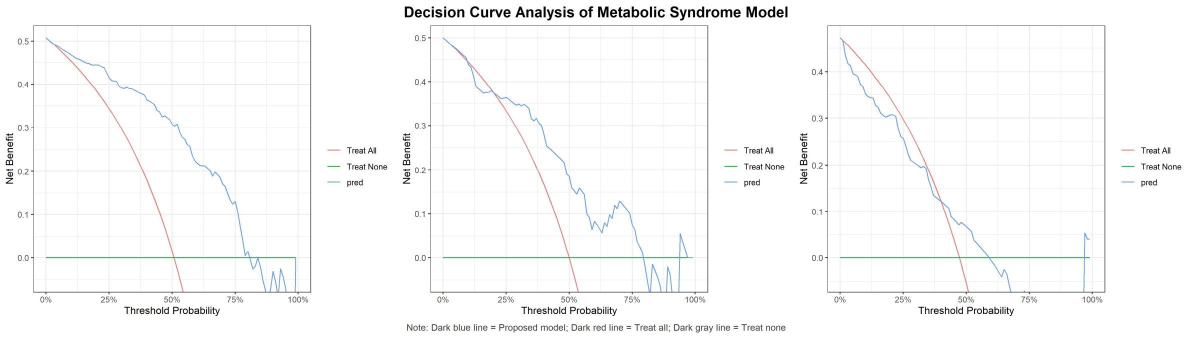

Supplement: Supplementary file 1 — Supplementary figures. [file ijmsv23p1645s1.pdf]
